# Observational Comparison of Outcomes of Sandplay Therapy (SPT-SAFE) Versus Dialectical Behavior Therapy (DBT-BI) for Elementary School Students with NSSI and Suicidal Ideation: A Retrospective School-Based Study

**DOI:** 10.3390/bs16020308

**Published:** 2026-02-23

**Authors:** Hyeonjeong Kwak, Unkyoung Ahn

**Affiliations:** 1 Department of Psychology, Dankook University, Cheonan 31116, Republic of Korea; kwakhj@dankook.ac.kr; 2 Institute for Mental-Health, Dankook University, 119 Dandae-ro, Cheonan 31116, Republic of Korea

**Keywords:** non-suicidal self-injury, suicidal ideation, school-based intervention, sandplay therapy, dialectical behavior therapy

## Abstract

Background/Objectives: Suicidal ideation and non-suicidal self-injury (NSSI) among elementary school students represent critical public health concerns that require develop-mentally appropriate, evidence-informed school-based interventions. This study con-ducted a retrospective comparative analysis of two school-based approaches—Sandplay Therapy with Suicidal Ideation and Self-Injury-Focused Engagement (SPT-SAFE) and a School-based Dialectical Behavior Therapy-informed Brief Intervention (DBT-BI)—for elementary school students presenting with suicidal ideation and NSSI. The objective was to describe pre–post-changes in key outcomes within each intervention and to explore whether outcome trajectories differed between the two approaches in a non-randomized, real-world school-based setting. Methods: This retrospective study analyzed archival clinical records from 109 elementary school students (SPT-SAFE: N = 59; DBT-BI: N = 50) who received services at a school-based suicide prevention center in South Korea between 2022 and 2024. Seven validated outcome measures assessed suicidal ideation, NSSI frequency, depression, anxiety, aggression, impulsiveness, and self-concept at pre- and post-intervention. Pre–post-changes and exploratory between-group differences were examined using 2 × 2 mixed-design ANOVAs (Group × Time interaction), with baseline-adjusted ANCOVAs conducted as complementary analyses. Suicidal ideation was operationalized using the SIQ-JR total score, and NSSI was operationalized using the FASM summed frequency index. Results: Both interventions were associated with significant reductions in suicidal ideation (F = 29.98, *p* < 0.001, partial η^2^ = 0.219) and NSSI frequency (F = 15.95, *p* < 0.001, partial η^2^ = 0.130), with large within-group effect sizes and no significant Group × Time interactions. Accordingly, between-group differences were limited and should be interpreted as exploratory rather than comparative–effectiveness evidence. Modest between-group differences favoring DBT-BI were observed for self-concept outcomes (F = 4.14, *p* = 0.044, partial η^2^ = 0.037; d = −0.39). Conclusions: These findings suggest that both interventions were associated with pre–post-improvements in suicidal ideation and NSSI frequency within a school-based clinical context.

## 1. Introduction

### 1.1. Background

Suicide represents one of the most urgent public health challenges affecting children and adolescents worldwide (Bridge et al., 2015; Nock et al., 2013). Among elementary school students (approximately 6–12 years old), the emergence of suicidal ideation and non-suicidal self-injury (NSSI) signals a critical developmental risk that warrants timely clinical attention (Duffy et al., 2018; Casey et al., 2008). In this study, suicidal ideation refers to self-reported thoughts about suicide (i.e., thinking about killing oneself), which are clinically relevant even when acute intent or an imminent plan is absent. In South Korea, suicide rates among children and adolescents have remained persistently elevated, with suicide consistently ranking among the leading causes of death in this age group (Statistics Korea, 2023; Liu, 2020).

NSSI—defined as the deliberate destruction of one’s own body tissue without suicidal intent—has emerged as a significant clinical concern among elementary school students (Klonsky et al., 2014). In a community sample, approximately 7.6% of third-graders reported lifetime NSSI, highlighting that NSSI can be present even in late childhood (Barrocas et al., 2012). Recent epidemiological evidence indicates that self-injury and suicidality can occur even in late childhood. In a cohort of 9–10-year-old children, DeVille et al. (2020) reported a notable prevalence of suicidal ideation, suicide attempts, and non-suicidal self-injury, with family-related factors associated with risk. Consistent with this, Van Hove et al. (2023) emphasize early-onset NSSI in elementary school-aged children, underscoring the importance of developmentally attuned assessment and intervention. Importantly, NSSI is among the strongest prospective predictors of subsequent suicide attempts, conferring a three- to four-fold increased risk even after controlling for other psychiatric risk factors (Ribeiro et al., 2016; Klonsky et al., 2014).

School-based mental health service delivery models have gained increasing recognition as effective platforms for identifying and supporting at-risk elementary school students (Fazel et al., 2014; Wyman et al., 2010). Such models offer several advantages, including reduced barriers to access, decreased stigma, integration with existing educational support systems, and the capacity to reach students who might not otherwise receive mental health services (Langley et al., 2010; Kataoka et al., 2007).

### 1.2. Evidence for Dialectical Behavior Therapy

Dialectical Behavior Therapy (DBT), originally developed for adults with borderline personality disorder and chronic suicidality (Linehan, 1993), has been adapted for adolescents (DBT-A) and has accumulated substantial empirical support for reducing NSSI and suicidal behaviors in youth populations (Mehlum et al., 2014; McCauley et al., 2018). Multiple randomized controlled trials have demonstrated the efficacy of DBT-based interventions for adolescents presenting with NSSI and suicidal behaviors (Kothgassner et al., 2021; Asarnow et al., 2017). Meta-analytic evidence further supports these findings; however, the underlying trials largely enrolled adolescents and older school-aged youth (approximately 11 to <18 years old), and evidence in younger elementary-aged children remains limited. A recent meta-analysis synthesizing a broad range of psychosocial interventions for self-harm in children and adolescents, including DBT-based approaches, reported overall beneficial effects on self-harm-related outcomes (Johansson et al., 2025). In addition, a network meta-analysis by Bahji et al. (2021) encompassing 44 randomized trials ranked DBT as the most effective intervention for adolescent NSSI (odds ratio = 0.28). Collectively, these findings position DBT as one of the most empirically supported treatments for suicidal and self-injurious behaviors in youths.

### 1.3. Evidence for Symbolic–Affective Approaches

Sandplay Therapy (SPT), grounded in Jungian analytical psychology and symbolic process theory, represents a therapeutic modality that emphasizes nonverbal and symbolic expression through the construction of miniature worlds in a sand tray (Kalff, 2003). Unlike cognitive–behavioral approaches, SPT primarily targets affective experience and meaning-making through symbolic representation rather than direct verbal instruction or skills training.

However, direct evidence for SPT specifically targeting NSSI outcomes remains limited, and most studies have primarily examined broader emotional and behavioral symptoms, with self-injury-related outcomes reported less consistently. Nevertheless, SPT may be developmentally congruent for youth with limited verbalization, offering a symbolic channel for affect processing relevant to NSSI-related distress. For example, Ahn and Kwak (2022) reported preliminary evidence suggesting reductions in depressive symptoms and suicidal ideation following an eight-session sandplay intervention among adolescents who had experienced suicide-related events. Furthermore, a recent meta-analysis by Wiersma et al. (2022), synthesizing 40 studies across eight countries (total N = 1284), reported large overall treatment effects (Hedges’ g ≈ 1.10) for emotional and behavioral symptoms, including internalizing and externalizing domains. Although suicidal outcomes were not directly examined, these findings highlight the potential of sandplay-based interventions for emotionally distressed youth. Beyond sandplay-specific interventions, complementary lines of research—including arts-enhanced DBT and mindfulness-based play therapy—suggest that the inclusion of expressive or symbolic components within evidence-based interventions may be associated with improved engagement and emotion regulation-related outcomes in youths (Smith et al., 2025). While these approaches differ in theoretical orientation and structure, they collectively highlight the potential relevance of symbolic and expressive processes for addressing intense affect and self-injurious behaviors in high-risk children.

### 1.4. Research Gap and Study Objectives

Despite the growing evidence base for both cognitive–behavioral approaches, such as DBT, and symbolic–affective approaches, such as SPT, to our knowledge, no published studies have directly compared these theoretically distinct intervention modalities in elementary school populations. This represents an important gap in the literature, as clinicians working in school-based settings are often required to make treatment selection decisions without empirical guidance regarding which approach may be more appropriate—or for which children—under real-world service conditions (Ougrin et al., 2015; Brent et al., 2013).

The present study seeks to address this gap by conducting a retrospective comparative analysis of two theoretically distinct, evidence-informed interventions for elementary school students presenting with suicidal ideation and NSSI: Sandplay Therapy with Suicidal Ideation and Self-Injury-Focused Engagement (SPT-SAFE) and a School-based Dialectical Behavior Therapy-informed Brief Intervention (DBT-BI). In the present study, we examined two school-based, developmentally adapted variants for youth presenting with suicidal ideation and/or NSSI. DBT-BI is a brief, individual intervention delivered in school settings that prioritizes targeted skills coaching and structured risk management, rather than applying the full multicomponent DBT package (e.g., individual therapy, group skills training, and telephone coaching) as-is. However, core DBT risk-management elements were retained, including ongoing diary card-based monitoring, prioritization (hierarchizing) of target risk behaviors, and session-centered chain analysis to conduct functional assessment and guide selective skills practice. SPT-SAFE builds on the symbolic–affective, experiential foundations of traditional Sandplay Therapy while explicitly integrating sustained clinical attention to suicidal ideation/NSSI risk within routine school-based care (e.g., ongoing risk monitoring and coordination pathways). These adaptations were implemented to enhance feasibility and engagement for upper-elementary students in real-world school-based services. Both interventions were delivered as part of routine clinical care at a school-based suicide prevention center commissioned by the Chungcheongnam-do Office of Education in South Korea.

The specific objectives of this study were to: (1) compare pre–post-changes in suicidal ideation (SIQ-JR total score) and NSSI frequency (FASM summed frequency score) following SPT-SAFE versus DBT-BI; (2) examine differential effects across multiple outcome domains, including depression, anxiety, aggression, impulsiveness, and self-concept; (3) characterize the magnitude of within-group change using standardized effect size metrics; and (4) evaluate baseline equivalence between treatment groups to assess potential selection bias inherent in this non-randomized observational design.

## 2. Materials and Methods

### 2.1. Study Design

This study was conducted as a retrospective observational comparative effectiveness study based on archival clinical records collected during routine school-based mental health care. All data were derived from previously delivered interventions and standardized psychological assessments conducted for clinical purposes, not for prospective research. No participants were recruited, assigned, or treated specifically for this study. Given the retrospective and non-randomized nature of the study, all analyses were conducted for descriptive and exploratory purposes. No causal inferences regarding treatment effectiveness or comparative superiority were intended.

### 2.2. Setting and Participants

The present study utilized archival clinical records from a school-based suicide prevention center commissioned by the Chungcheongnam-do Office of Education in South Korea. As of 2024, Chungcheongnam-do comprised approximately 1243 elementary, middle, and high schools, serving an estimated total student population of about 250,000. The center has provided standardized crisis intervention services for students referred by schools due to concerns regarding suicidal ideation, NSSI, or related emotional and behavioral risks. During the retrospective study period (approximately 2022–2024), an average of 120–150 elementary school students per year were referred to the center for suicide- or NSSI-related concerns. Over this period, the cumulative number of elementary school referrals is estimated to be approximately 420 cases. Of these cases, only those meeting strict eligibility criteria and having complete pre- and post-intervention assessment data were included in the final analysis.

#### 2.2.1. Treatment Allocation and Clinical Decision-Making

Because this study retrospectively analyzed records collected during routine clinical care, treatment allocation was not randomized. Assignment to SPT-SAFE or DBT-BI followed routine clinical decision-making at the school-based suicide prevention center, based on standardized intake assessment and documentation. All referred students received an initial suicide risk assessment, clinical interview, and caregiver consultation. Students with acute suicide intent, imminent suicide plans, or a recent medically serious suicide attempt were excluded from both interventions and referred to higher-intensity services. Among eligible students with suicidal ideation and/or NSSI, treatment selection reflected routine clinical considerations, including developmental and communication factors, intake-documented clinical and contextual information (e.g., prior psychiatric treatment history and school-related stressors such as bullying exposure), caregiver preference, and therapist availability within each treatment track. In addition, assignment considered operational balance across treatment tracks (e.g., case-mix and therapist caseload) to avoid a systematic concentration of higher-severity cases in a single track. Treatment allocation was determined as part of routine care, independent of the present study and prior to outcome evaluation. Baseline characteristics documented at intake are summarized in Table 1, and standardized mean differences (SMDs) are additionally reported to describe baseline balance under the non-randomized design (largest |SMD| = 0.31 for SES; bullying exposure |SMD| = 0.06). Accordingly, although baseline characteristics were described and compared, residual confounding inherent to non-randomized allocation cannot be fully excluded.

#### 2.2.2. Inclusion Criteria

Cases were eligible for inclusion based on a retrospective review of clinical records if the following criteria were documented: (1) elementary school students (Grade 4 or above) with sufficient literacy to complete self-report measures in Korean; (2) documented NSSI within the six months prior to intake; (3) presence of suicidal ideation without acute intent or imminent plan, as recorded in the Columbia Suicide Severity Rating Scale (C-SSRS); (4) availability of both pre- and post-intervention psychological assessment data; (5) documented consent for clinical services from both the students and caregiver; and (6) receipt of at least six therapeutic sessions.

#### 2.2.3. Exclusion Criteria

Cases were excluded from the analysis based on retrospective review of clinical records if any of the following conditions were documented: (1) clear suicidal intent or an active suicide plan requiring inpatient admission or emergency medical care; (2) severe psychiatric conditions necessitating immediate pharmacological intervention or intensive psychiatric treatment; (3) recent suicide attempts involving significant medical injury; (4) emotional distress cases without a documented history of NSSI; (5) documented premature termination of treatment; (6) receipt of fewer than six therapy sessions; or (7) transfer to inpatient or intensive treatment during the treatment episode.

#### 2.2.4. Final Sample

**Figure 1:**
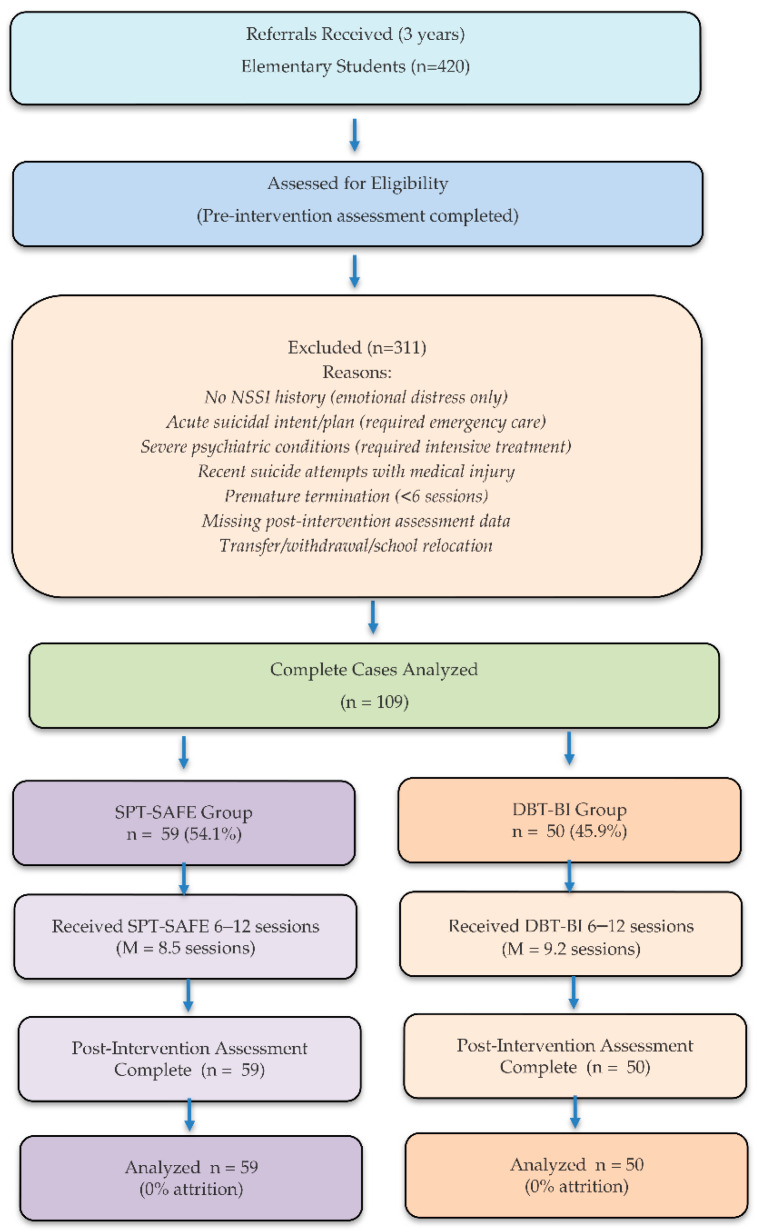
Flow diagram. Treatment allocation was based on standardized clinical decision rules rather than randomization.

After applying eligibility criteria and excluding incomplete cases, the final analytical sample consisted of 109 elementary school students, including 59 in the SPT-SAFE group (54.1%) and 50 in the DBT-BI group (45.9%). Figure 1 illustrates the case selection and exclusion process in accordance with STROBE recommendations for observational studies (von Elm et al., 2007).

### 2.3. Interventions

#### 2.3.1. Common Initial Procedures

As part of routine clinical practice, all referred students underwent standardized initial procedures before any therapeutic intervention: (1) suicide risk assessment using the Columbia Suicide Severity Rating Scale (C-SSRS; Posner et al., 2011); (2) Safety Planning Intervention (SPI) development (Stanley & Brown, 2012); and (3) caregiver and school coordination for crisis response planning. These procedures were implemented independently of the present study and uniformly applied across intervention types.

#### 2.3.2. SPT-SAFE

The core intervention phase (approximately 8–12 sessions, M = 8.5 sessions) emphasized: (1) nonverbal and symbolic expression through sandplay; (2) bottom-up affect regulation (body → emotion → cognition); (3) a therapeutic sequence of symbolization, meaning-making, and relational regulation; (4) explicit and ongoing clinical attention to suicide and NSSI risk throughout the therapeutic process; and (5) an emphasis on emotional stabilization and safety, with interpretive work intentionally limited when risk levels were elevated.

Consistent with the SPT-SAFE framework, suicide risk assessment and safety management procedures were informed by established evidence-based approaches, including Dialectical Behavior Therapy (DBT), the Collaborative Assessment and Management of Suicidality (CAMS), and Brief Cognitive Behavioral Therapy (BCBT). These elements were incorporated as safety-focused clinical procedures rather than skills-training interventions and included ongoing risk monitoring, brief stabilization strategies (e.g., breathing or grounding exercises), and behavioral observation of affective dysregulation during sessions.

Non-directivity was preserved with respect to symbolic expression and scene construction, while safety-related procedures were implemented at the level of the clinical process.

#### 2.3.3. DBT-BI

The DBT-BI applied in the present study was not a full Dialectical Behavior Therapy for Adolescents (DBT-A) protocol, but a developmentally adapted, school-based, DBT-informed brief intervention designed for elementary school students with suicidal ideation and NSSI. Accordingly, the intervention focused on selected core DBT components (e.g., behavioral monitoring, chain analysis, and targeted skills practice) delivered in an individualized, time-limited format without skills groups or between-session coaching.

The intervention consisted of approximately 8–12 sessions (M = 9.2 sessions) and focused on the following components, applied in a developmentally and clinically sequenced manner: (1) brief mindfulness-based stabilization at the beginning of sessions; (2) systematic monitoring of suicidal behaviors and NSSI using diary cards; (3) risk behavior analysis using a DBT-based chain analysis of suicidal and NSSI behaviors; (4) collaborative practice of skills targeting identified risk behaviors, with emphasis on emotion regulation and distress tolerance; and (5) impulse control strategies and structured, goal-oriented skill acquisition within a time-limited treatment format.

Sessions followed a consistent clinical sequence. Each session typically began with brief mindfulness-based stabilization, followed by a diary card review and DBT-based chain analysis to identify priority risk targets. In later sessions, selected DBT skills were collaboratively practiced to address identified target behaviors, consistent with DBT principles of validation, dialectical balance, and behavior change, with skill selection individualized based on ongoing behavioral assessment and clinical judgment.

#### 2.3.4. Therapist Structure, Team Separation, and Supervision

To prevent cross-contamination between treatment models, SPT-SAFE and DBT-BI were delivered by separate therapist teams with no overlap in providers. Each intervention was implemented by a specialized team of licensed clinical or counseling psychologists. Therapists within each treatment condition treated multiple students exclusively within a single intervention model.

Therapists received regular model-specific supervision, including weekly or biweekly case consultation meetings, focusing on adherence to core intervention principles and clinical risk management.

### 2.4. Outcome Measures

A comprehensive battery of seven validated self-report measures was administered at pre-intervention (baseline) and post-intervention (after 6–12 sessions). All measures used Korean-validated versions with established psychometric properties. The assessment completion rate was 100% (N = 109; no missing data). For all measures, total scores were computed according to each instrument’s scoring guidelines.

#### 2.4.1. Primary Outcomes

Suicidal Ideation Questionnaire—Junior (SIQ-JR; Reynolds, 1988): 15-item measure assessing suicidal ideation frequency and intensity on a 7-point Likert scale (0–6), administered at both pre-intervention (baseline) and post-intervention assessments. Score range: 0–90. Higher scores indicate greater suicidal ideation. Korean version Cronbach’s α = 0.89–0.93 (Y. S. Lee et al., 2004).

Functional Assessment of Self-Mutilation (FASM; Lloyd et al., 1997): A self-report measure assessing NSSI behaviors and their associated functions. Includes a checklist of approximately 11 common NSSI methods. Assesses the frequency of each NSSI method over the past year using a 7-point Likert scale (0 = none to 6 = six or more times). Higher summed frequency scores and higher functional subscale scores indicate greater NSSI frequency and stronger endorsement of NSSI-related functions. The Korean version demonstrated good internal consistency (Cronbach’s α = 0.85) (Kwon & Kwon, 2017). For analysis, we computed an overall NSSI frequency index by summing method-specific frequency ratings; thus, the primary FASM outcome reflects NSSI frequency.

#### 2.4.2. Secondary Outcomes

Center for Epidemiologic Studies Depression Scale for Children (CES-DC; Radloff, 1977): 20-item measure assessing depressive symptoms on a 4-point scale (0–3). Score range: 0–60. Higher scores indicate more severe depressive symptoms. Korean version Cronbach’s α = 0.89 (Cho & Lee, 1990).

State-Trait Anxiety Inventory for Children—Trait Version (STAI-T; Spielberger, 1973): 20-item measure assessing stable anxiety tendency on a 3-point scale (1–3). Score range: 20–60. Higher scores indicate higher trait anxiety. Korean version Cronbach’s α = 0.86–0.88 (Cho & Choi, 1989).

Aggression Questionnaire (AQ; Buss & Perry, 1992): 27-item measure assessing aggression and hostility on a 5-point Likert scale (1–5). Score range: 27–135. Higher scores indicate greater aggression. Korean version Cronbach’s α = 0.86–0.91 (Seo & Kwon, 2002).

Barratt impulsivity Scale (BIS-11; Patton et al., 1995): 23-item shortened version assessing impulsivity on a 4-point scale (1–4). Score range: 23–92. Higher scores indicate greater impulsivity. Korean version Cronbach’s α = 0.78–0.82 (S. Y. Lee et al., 2012).

Piers–Harris Children’s Self-Concept Scale (PHCSCS; Piers, 2022): Self-concept was assessed using a modified short-form version derived from the Piers–Harris Children’s Self-Concept Scale, Second Edition (PHCSCS-2). Thirty items were selected from the original item pool, corresponding to the Korean-translated PHCSCS items, and the response format was adapted from the original dichotomous (Yes/No) format to a 6-point Likert scale to enhance sensitivity. This modified version has been used in prior research with school-aged populations (Ramanathan et al., 2020). In the present sample, the modified self-concept scale demonstrated good internal consistency (Cronbach’s α = 0.88). Scores were computed by summing item responses; higher scores indicate more positive self-concept (i.e., improvement is reflected by score increases).

### 2.5. Statistical Analysis

All statistical analyses were conducted using IBM SPSS Statistics (version 25) in accordance with standard procedures for observational studies.

Baseline homogeneity testing: Independent-sample *t*-tests were conducted to assess baseline equivalence between SPT-SAFE and DBT-BI groups on all seven outcome measures. Assumptions were checked (Shapiro–Wilk; Levene’s test), and Welch’s *t*-test was used when variance homogeneity was not met. Baseline tests were descriptive (sample characterization), so multiplicity correction was not applied; the findings were interpreted with effect magnitude/clinical relevance. Statistical significance was set at α = 0.05 (two-tailed).

Primary analysis: A 2 × 2 mixed-design ANOVA was conducted for each outcome measure with Group (SPT-SAFE vs. DBT-BI) as the between-subjects factor and Time (pre-intervention vs. post-intervention) as the within-subjects factor. Effects of interest included: (1) main effect of Time (overall pre-post change); (2) main effect of Group (overall group difference); and (3) Group × Time interaction (differential treatment effects). The prespecified primary inferential test for differential pre–post-change was the Group × Time interaction. Statistical significance was set at α = 0.05. Effect size was calculated using partial eta-squared (partial η^2^): small = 0.01, medium = 0.06, and large = 0.14.

Secondary analysis: One-way ANCOVA was conducted for each outcome measure with Group as the independent variable, post-intervention score as the dependent variable, and pre-intervention score as the covariate ANCOVA was conducted as a complementary baseline-adjusted (sensitivity/robustness) analysis; if results diverged, inference prioritized the prespecified interaction model. Statistical significance was set at α = 0.05.

Effect sizes: Within-group effect sizes (pre-post-change) were calculated using Cohen’s d for paired samples. Between-group effect sizes (group comparison on change scores) were calculated using Cohen’s d for independent samples. Interpretation: small = 0.20, medium = 0.50, and large = 0.80.

Missing data: Outcome assessments were complete at both timepoints, analyses were effectively complete-case by design. Only cases with complete pre- and post-intervention data on all seven outcome measures were included (N = 109). No imputation methods were used.

### 2.6. Ethical Considerations

This study was conducted in accordance with the Declaration of Helsinki and approved by the Institutional Review Board (IRB) of Dankook (IRB No: DKU 2025-12-004, Date: 11 December 2025). All students and their caregivers had provided written informed consent for clinical services at the time of treatment. For this retrospective analysis of de-identified archival data, a waiver of additional informed consent was granted by the IRB. All data were de-identified prior to analysis, stored securely on encrypted devices, and accessed only by authorized research personnel.

## 3. Results

### 3.1. Participant Characteristics

The final analytical sample consisted of 109 elementary school students, including 59 in the SPT-SAFE group (54.1%) and 50 in the DBT-BI group (45.9%). Participant demographic and characteristics are presented in Table 2. Both groups were predominantly female (SPT-SAFE: 59.3%; DBT-BI: 60.0%). Mean age was 11.41 ± 0.77 years in the SPT-SAFE group and 11.68 ± 1.20 years in the DBT-BI group. Treatment duration was comparable between groups (SPT-SAFE: M = 8.5 sessions; DBT-BI: M = 9.2 sessions).

### 3.2. Baseline Homogeneity Testing

Independent samples t-tests indicated no significant baseline differences between the SPT-SAFE and DBT-BI groups across any of the seven outcome measures (all *p* > 0.05), supporting baseline comparability at the level of primary and secondary outcomes (Table 3). Baseline effect sizes (Cohen’s d) ranged from −0.23 to 0.18, all within the negligible range. As shown in Table 1, the two groups were broadly comparable across multiple clinical and contextual variables relevant to treatment allocation, including baseline symptom severity, NSSI frequency, socioeconomic status, and behavioral dysregulation. Notable differences were observed for prior psychiatric treatment history and bullying exposure, reflecting routine clinical triage considerations in a non-randomized assignment process.

### 3.3. Primary Outcomes: Mixed ANOVA Results

#### 3.3.1. Suicidal Ideation (SIQ-JR)

A significant main effect of Time was observed for suicidal ideation (F(1,107) = 29.98, *p* < 0.001, partial η^2^ = 0.219; large), indicating substantial reductions from pre- to post-intervention across both groups. No significant main effect of Group was observed (F(1,107) = 0.74, *p* = 0.393, partial η^2^ = 0.007; small). No significant Group × Time interaction was observed for suicidal ideation (F(1,107) = 0.30, *p* = 0.584, partial η^2^ = 0.003; small), indicating no statistically significant difference in change between interventions.

#### 3.3.2. NSSI (FASM)

A significant main effect of Time was also observed for NSSI (F(1,107) = 15.95, *p* < 0.001, partial η^2^ = 0.130; large), reflecting overall reductions in NSSI frequency, as indexed by summed frequency scores on FASM across groups. Neither the main effect of Group (F(1,107) = 2.10, *p* = 0.150, partial η^2^ = 0.019; small) nor the Group × Time interaction (F(1,107) = 1.31, *p* = 0.255, partial η^2^ = 0.012; small) reached statistical significance.

### 3.4. Secondary Outcomes: Mixed ANOVA Results

Significant main effects of Time were observed for depressive symptoms (CES-DC: F(1,107) = 18.02, *p* < 0.001, partial η^2^ = 0.144; large), anxiety (STAI-T: F(1,107) = 9.97, *p* = 0.002, partial η^2^ = 0.085; medium), and aggression (AQ: F(1,107) = 8.11, *p* = 0.005, partial η^2^ = 0.070; medium), indicating improvements across both intervention groups. No significant change over time was observed for impulsivity (BIS: F(1,107) = 0.10, *p* = 0.757, partial η^2^ = 0.001; small).

**Figure 2:**
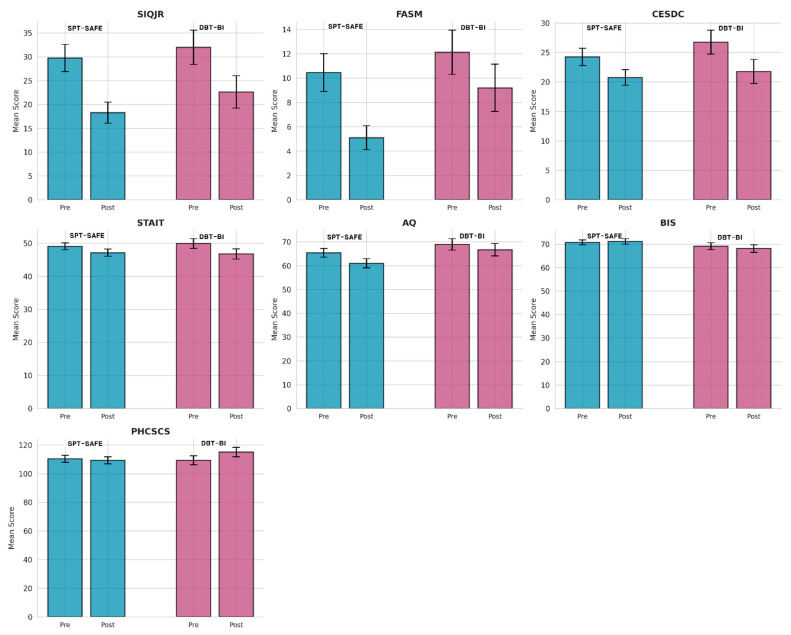
Pre–Post-comparison charts for all outcome measures. Note. Higher scores on the PHCSCS indicate a more positive self-concept, whereas higher scores on all other measures indicate greater symptom severity.

**Figure 3:**
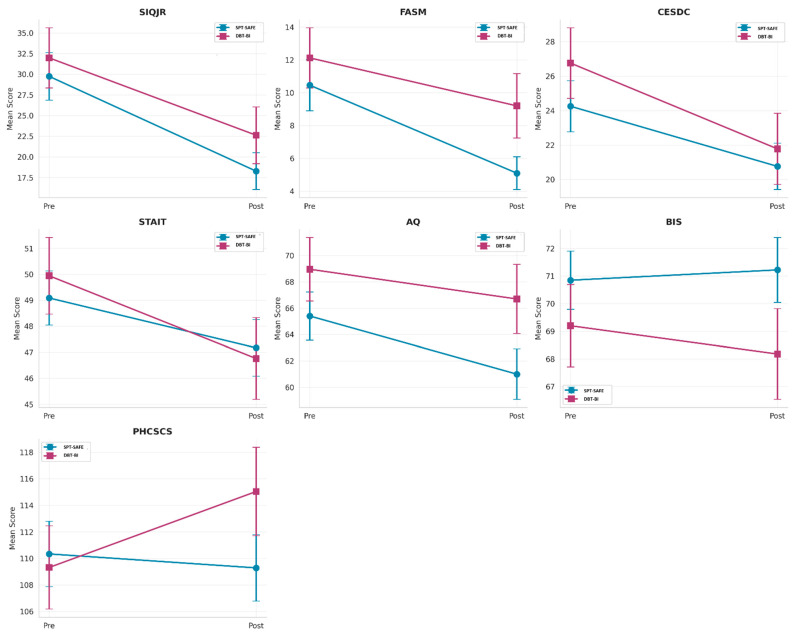
Group × Time interaction plots. Note. Higher scores on the PHCSCS indicate a more positive self-concept, whereas higher scores on all other measures indicate greater symptom severity.

A significant Group × Time interaction was identified for self-concept (PHCSCS: F(1,107) = 4.14, *p* = 0.044, partial η^2^ = 0.037; small). Post hoc inspection indicated greater improvement in self-concept scores in the DBT-BI group relative to the SPT-SAFE group. The SPT-SAFE group showed no statistically significant change in self-concept, whereas the DBT-BI group demonstrated a modest increase. Consistent with this interaction, within-group post hoc tests indicated no significant change in the SPT-SAFE group (*p* = 0.638; d = −0.06), whereas the DBT-BI group showed a modest increase (*p* = 0.026; d = 0.26). A summary of the mixed-design ANOVA results for all outcome measures is provided in Table 4. Pre–post-changes across all outcome measures for both intervention groups are illustrated in Figure 2. Group × Time interaction patterns for the outcome measures, including self-concept, are visualized in Figure 3.

### 3.5. ANCOVA Results

One-way ANCOVAs controlling for baseline scores corroborated the mixed ANOVA findings. A significant group difference emerged only for self-concept, with higher baseline-adjusted post-intervention scores in the DBT-BI group compared to the SPT-SAFE group (F(1,106) = 4.33, *p* = 0.040, partial η^2^ = 0.039; small). No other outcome variables demonstrated statistically significant group differences after adjustment for baseline scores (all *p* > 0.05). The results of the ANCOVA analyses controlling for baseline scores are presented in Table 5.

### 3.6. Effect Size Analyses

Within-group effect size analyses (Cohen’s d) in this section quantify within-group pre–post-change and between-group differences in change scores (Table 6), whereas partial η^2^ values are reported for the mixed ANOVA (Table 4) and ANCOVA (Table 5) results above. Within-group effect size analyses (Cohen’s d) indicated moderate-to-large pre–post-reductions in suicidal ideation and NSSI in both intervention groups (Table 6). For the SPT-SAFE group, effect sizes were largest for suicidal ideation (d = −0.52) and NSSI (d = −0.45). For the DBT-BI group, comparable reductions were observed for suicidal ideation (d = −0.36) and NSSI (d = −0.39). Moderate within-group effects were also observed for depressive and anxiety symptoms in both groups.

**Figure 4:**
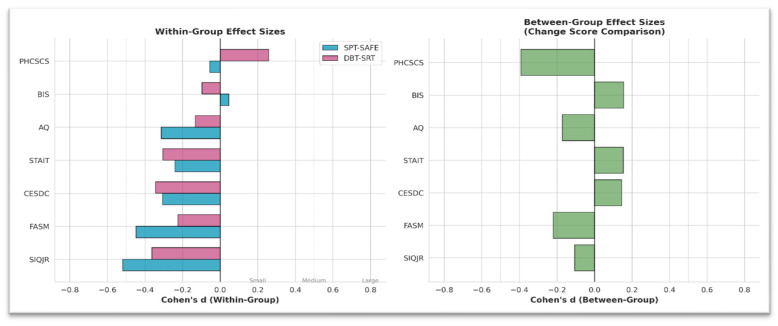
Effect sizes comparison.

Between-group effect sizes for change scores were small across all outcomes, with the exception of self-concept (d = −0.39, 95% CI [−0.77, −0.01]), favoring the DBT-BI group. No other between-group differences reached statistical significance. Within-group and between-group effect size estimates are summarized in Table 6. A visual comparison of within-group and between-group effect sizes across outcomes is presented in Figure 4.

## 4. Discussion

### 4.1. Summary of Principal Findings

In this retrospective observational study, both interventions were associated with significant pre–post-reductions in suicidal ideation and reductions in NSSI frequency, alongside improvements in several secondary domains (e.g., depression, anxiety, and aggression). Across most outcomes, the analyses provided no statistically significant evidence of differential change between groups. These findings indicate that no statistically detectable between-group differences were observed in outcome trajectories, within the limits of the retrospective observational design and available statistical power. A small Group × Time effect favoring DBT-BI emerged for self-concept, while other between-group differences were not detected after adjustment for baseline scores. The absence of statistically significant Group × Time interactions should not be interpreted as evidence of formal equivalence between interventions, but rather as an absence of detected between-group differences within the constraints of a retrospective observational design.

### 4.2. Interpretation of Findings

#### 4.2.1. Similar Improvement in Core Safety Outcomes

The absence of significant Group × Time differences for suicidal ideation and NSSI-related indices suggests that, within the constraints of the present study, the two theoretically distinct approaches—one emphasizing bottom-up symbolic–affective processing (SPT-SAFE) and the other emphasizing top-down skills-based behavior change (DBT-BI)—did not demonstrate statistically distinguishable patterns of change in key safety-relevant outcomes within a school-based service context. Importantly, given the observational design, these findings should be interpreted as indicating no detected between-group differences in change rather than formal equivalence.

This pattern is broadly consistent with the common factors literature in psychotherapy, which posits that shared therapeutic elements (e.g., therapeutic alliance, hope, emotional engagement, and systematic attention to problems) account for a substantial proportion of outcome variance across treatment modalities (Wampold, 2015; Cuijpers et al., 2019). However, common-factor constructs such as therapeutic alliance and expectancy were not directly measured in the present study.

Several evidence-based elements were implemented across both interventions and may have contributed to observed improvements: (1) structured safety planning prior to intervention-specific treatment using the Safety Planning Intervention (Stanley & Brown, 2012); (2) coordination with caregivers and schools for crisis response planning; (3) an explicit focus on emotion regulation, implemented via different therapeutic mechanisms; and (4) consistent therapeutic contact within a time-limited format.

The within-group effect sizes observed for suicidal ideation reduction in both interventions (SPT-SAFE: d = −0.52; DBT-BI: d = −0.36) compare favorably with meta-analytic benchmarks reported for youth psychosocial interventions targeting suicidal ideation and NSSI. Glenn et al. (2019) reported an average effect size of d = 0.34 for psychosocial treatments targeting suicidal ideation in youth, while Ougrin et al. (2015) reported pooled effects of d = 0.34 for interventions targeting NSSI. The present findings exceed these benchmarks, although such comparisons should be interpreted cautiously given the differences in the study design and measurement.

#### 4.2.2. Differential Effect on Self-Concept

The finding that DBT-BI was associated with a greater improvement in self-concept than SPT-SAFE was contrary to the initial hypothesis. One plausible explanation is that DBT-BI’s structured skills-training format may foster a sense of competence and mastery through repeated practice and successful application of concrete coping strategies. According to Bandura’s self-efficacy theory, mastery experiences are the most influential source of self-efficacy beliefs, which in turn contribute to positive self-concept development (Bandura, 1977). Given the exploratory nature of this finding and the absence of correction for multiple comparisons, this result should be interpreted with caution.

Even as a brief, school-based intervention, DBT-BI may facilitate experiential engagement in DBT skills that is associated with improvements in functioning and behavioral control, potentially strengthening self-efficacy and contributing to more positive self-concept development (Neacsiu et al., 2010; Mazzucchelli et al., 2010). For elementary school students in the concrete operational stage of cognitive development (Piaget, 1969), such observable skill acquisition may be more readily integrated into self-concept than the implicit symbolic meaning-making emphasized in sandplay-based approaches.

It is important to note that the magnitude of this differential effect was small (d = −0.39; partial η^2^ = 0.037), and both groups demonstrated improvements in depression and anxiety, constructs closely related to self-concept (Sowislo & Orth, 2013).

### 4.3. Comparison to the Existing Literature

#### 4.3.1. DBT Evidence Base and Developmental Extension

The present findings align with and extend the existing evidence base for DBT-informed interventions in reducing NSSI and suicidal ideation among youths. The within-group effect size for suicidal ideation reduction observed in the DBT-BI group (d = −0.36) is comparable to the controlled effect size reported in the meta-analysis by Kothgassner et al. (g = −0.31) across 21 studies of DBT-based interventions (Kothgassner et al., 2021).

Most prior DBT-A trials have focused on adolescents aged 13–18 years (Mehlum et al., 2014; McCauley et al., 2018; Goldstein et al., 2015), whereas the present study examined elementary school students, thereby extending the developmental scope of DBT-informed interventions. Moreover, standard DBT-A protocols typically involve comprehensive and intensive treatment over 16–24 weeks, including skills groups and phone coaching (Miller et al., 2007). In contrast, the present DBT-BI model consisted of 8–12 individual sessions delivered in a school-based setting. The observation of meaningful pre–post-improvements under this lower-intensity format suggests potential feasibility for school-based implementation.

#### 4.3.2. Symbolic–Affective and Sandplay-Related Evidence

The SPT-SAFE intervention applied in the present study can be conceptualized as an integration of traditional Sandplay Therapy’s symbolic–affective framework with sustained clinical attention to suicide and NSSI risk. For children and adolescents who exhibit limited verbal expression or difficulties in emotional awareness and regulation, non-directive symbolic expression has been proposed as a developmentally congruent pathway for accessing, externalizing, and modulating internal experiences associated with suicidal ideation and NSSI (Ahn & Kwak, 2022; Wiersma et al., 2022).

SPT-SAFE may plausibly influence suicidal ideation and NSSI through three complementary pathways: (A) a symbolic–narrative (meaning-making) pathway, in which sandplay supports the externalization and organization of distress into a shareable symbolic story; (B) a sensory–somatic (bottom-up) pathway, in which tactile, sensory–motor engagement facilitates arousal modulation and affect processing when verbal labeling is limited; and (C) a relational co-regulation pathway, in which empathic witnessing within a “free and protected space” strengthens therapeutic safety and engagement, supporting regulation in the context of connection.

Emerging evidence further suggests that creative and expressive approaches may contribute to emotional regulation and self-understanding in populations at risk for NSSI. For example, Smith et al. (2025) reported that an intervention integrating creative arts with dialectical behavior therapy was associated with clinically meaningful emotional and functional improvements among adolescents and young adult women with histories of NSSI. Taken together, these findings indicate that symbolic–affective interventions incorporating explicit risk awareness may play a clinically meaningful role in addressing emotional distress and NSSI-related difficulties among children and adolescents with suicidal ideation and NSSI, extending beyond their traditional use as general emotion-focused therapies.

These findings are consistent with symbolic–affective change processes in sandplay-based work. However, because the present study is retrospective, mechanisms cannot be evaluated directly and should be tested in future prospective research using potential mediators such as emotion regulation, distress tolerance, and relational safety.

### 4.4. Clinical Implications

The present findings indicate that both SPT-SAFE and DBT-BI represent viable school-based intervention options for elementary school students presenting with suicidal ideation and NSSI. The comparable effectiveness of the two interventions on core safety outcomes suggests that multiple therapeutic pathways can be effective within school settings.

SPT-SAFE may warrant consideration for students with limited verbal expression or heightened emotional arousal, for whom non-directive symbolic expression and bottom-up affect regulation facilitate emotional access, containment, and stabilization while maintaining ongoing clinical attention to suicide and NSSI risk. In contrast, DBT-BI may warrant consideration for students who demonstrate sufficient cognitive and emotional readiness to engage in behavioral risk analysis, structured skills practice, and therapeutic feedback. Improvements in self-concept observed in the DBT-BI group may reflect downstream effects of mastery experiences and enhanced self-efficacy rather than primary treatment targets. These treatment-matching considerations were not tested in this retrospective observational study and should be interpreted as hypothesis-generating pending prospective confirmation.

The brief format of both interventions (8–12 sessions) enhances feasibility within academic calendars and school scheduling constraints, while school-based delivery may reduce barriers related to access, stigma, and logistical burden for at-risk students (Fazel et al., 2014; Langley et al., 2010).

### 4.5. Theoretical Implications

The comparable effectiveness observed across theoretically distinct interventions raises questions regarding mechanisms of change. From a common factors perspective, shared therapeutic elements may account for much of the observed improvement (Wampold, 2015; Cuijpers et al., 2019). Alternatively, a specific mechanisms perspective suggests that different therapeutic pathways—symbolic–affective integration versus cognitive–behavioral skill acquisition—may lead to similar clinical endpoints through distinct processes (Kazdin, 2007; Laurenceau et al., 2007).

The comparison of DBT-BI (top-down, cognitive–behavioral) and SPT-SAFE (bottom-up, symbolic–affective) approaches also provides an empirical context for examining different models of emotion regulation development. Top-down models emphasize cognitive control and reappraisal (Gross, 1998; Ochsner & Gross, 2005), whereas bottom-up models emphasize somatic and subcortical emotional processing through sensory–motor and symbolic engagement (Schore, 1994; Porges, 2001). The present findings suggest that multiple regulatory pathways may be effective in reducing emotional distress in high-risk elementary school students.

### 4.6. Strengths and Limitations

This study has several notable strengths. First, it employed a real-world school-based effectiveness design, enhancing external validity. Second, the use of an active comparison between two theoretically distinct interventions provided a more rigorous test than usual care designs. Third, baseline characteristics were broadly comparable between groups despite non-randomized allocation. Fourth, this study focused on elementary school students with suicidal ideation and NSSI, an understudied yet clinically important population. Finally, the sample size (N = 109) is relatively large for a school-based effectiveness study.

Several limitations of the present study should be acknowledged. First, the retrospective observational design precludes causal inference. Second, the absence of long-term follow-up limits conclusions regarding the durability of treatment effects. Third, all outcomes relied on youth self-report measures. Fourth, because the cohort comprised students referred for suicidal ideation and/or NSSI, we could not separately evaluate SI-only and NSSI-only presentations, limiting presentation-specific inference. Fifth, although the sample size is reasonable for a school-based effectiveness study, power to detect small between-group effects may be limited; moreover, under non-randomized allocation, baseline differences (e.g., prior psychiatric treatment and bullying exposure) may reflect residual confounding despite baseline-adjusted analyses.

With respect to measurement, NSSI outcomes were indexed using the FASM, a validated measure that captures both the range of NSSI methods and their reported frequency. However, the FASM does not provide fine-grained temporal frequency estimates, which should be considered when interpreting the observed changes (Lloyd et al., 1997).

Future research should prioritize prospective randomized controlled trials with longer-term follow-up to evaluate maintenance of gains and relapse/recurrence in suicidal ideation and NSSI. Studies incorporating presentation-type stratification (SI-only vs. NSSI-only) and mechanism-focused measures (e.g., emotion regulation, therapeutic engagement, and symbolic–affective processing) would clarify how sandplay-based approaches exert effects in school-based services.

## 5. Conclusions

This retrospective comparative effectiveness study provides the first direct evidence comparing a symbolic–affective intervention (SPT-SAFE) and a cognitive–behavioral intervention (DBT-BI) for elementary school students with suicidal ideation and NSSI. Both interventions were associated with significant reductions in suicidal ideation and NSSI. Across most outcome domains, no statistically detectable between-group differences were observed, within the limits of the retrospective design and available statistical power. A modest differential effect favoring DBT-BI was observed for self-concept improvement, suggesting potential relevance for personalized treatment matching based on specific clinical needs.

These findings support the feasibility and clinical utility of both SPT-SAFE and DBT-BI as school-based suicide prevention interventions. Implemented in brief formats within real-world educational settings, both approaches demonstrated feasibility and were associated with within-group improvements, although this study provides an initial retrospective observational comparison rather than definitive comparative evidence.

From a broader perspective, the comparable outcomes observed across theoretically distinct interventions suggest that multiple pathways—symbolic–affective and cognitive–behavioral—may lead to meaningful reductions in suicidal ideation and self-injury. Future prospective randomized controlled trials with long-term follow-up and mechanism-focused analyses are needed to clarify for whom and through which processes these interventions exert their effects.

## Figures and Tables

**Table 1:** Baseline clinical and contextual characteristics used in treatment allocation (with SMD).

Domain	Variable	SPT-SAFE (N = 59)	DBT-BI (N = 50)	SMD
Demographics	Age (years)	11.41 ± 0.77	11.68 ± 1.20	0.27
	Male sex (%)	40.70%	40.00%	0.01
Clinical severity	SIQ-JR (baseline)	29.75 ± 22.15	31.98 ± 25.75	0.09
	CES-DC (baseline)	24.25 ± 11.40	26.76 ± 14.48	0.19
	STAI-T (baseline)	49.08 ± 7.98	49.94 ± 10.44	0.09
NSSI frequency	FASM (baseline)	10.46 ± 11.97	12.12 ± 12.97	0.19
Behavioral dysregulation	AQ (baseline)	65.41 ± 14.07	68.96 ± 17.10	0.23
	BIS (baseline)	70.85 ± 8.10	69.20 ± 10.54	−0.18
Family/SES	SES (1 = High, 3 = Low)	1.79 ± 0.64	1.97 ± 0.50	0.31
Prior treatment	Prior psychiatric treatment (%)	32.20%	40.80%	0.18
School factors	Bullying exposure (%)	11.90%	14.00%	0.06
Health	BMI	20.26 ± 4.72	20.64 ± 5.25	0.08

Note. Values are mean ± SD or percentage. SES coded such that higher scores indicate a lower socioeconomic status. Absolute SMDs were interpreted as follows: <0.10 negligible, 0.10–0.20 small, 0.20–0.50 moderate, and >0.50 large.

**Table 2:** Participant demographic characteristics.

Characteristic	SPT-SAFE (N = 59)	DBT-BI (N = 50)	Total (N = 109)
Age M (SD)	11.41 (0.77)	11.68 (1.20)	11.53 (0.99)
Male N (%)	24 (40.7%)	20 (40.0%)	44 (40.4%)
Female N (%)	35 (59.3%)	30 (60.0%)	65 (59.6%)

**Table 3:** Baseline equivalence testing results.

Variable	t	df	*p*	Cohen’s d	Baseline Difference
SIQJR	−0.487	107	0.627	−0.094	Not significant
FASM	−0.695	107	0.488	−0.134	Not significant
CESDC	−1.01	107	0.315	−0.194	Not significant
STAIT	−0.484	107	0.629	−0.093	Not significant
AQ	−1.191	107	0.236	−0.229	Not significant
BIS	0.922	107	0.359	0.177	Not significant
PHCSCS	0.259	107	0.796	0.05	Not significant

Conclusion: All groups were homogeneous at baseline (all *p* > 0.05).

**Table 4:** Mixed ANOVA results for all outcome measures.

Variable	Group Effect	Time Effect	Interaction Effect
SIQ-JR	F = 0.735, *p* = 0.393, partial η^2^ = 0.007	F = 29.981, *p* < 0.001, partial η^2^ = 0.219	F = 0.302, *p* = 0.584, partial η^2^ = 0.003
FASM	F = 2.104, *p* = 0.150, partial η^2^ = 0.019	F = 15.954, *p* < 0.001, partial η^2^ = 0.130	F = 1.308, *p* = 0.255, partial η^2^ = 0.012
CES-DC	F = 0.624, *p* = 0.431, partial η^2^ = 0.006	F = 18.023, *p* < 0.001, partial η^2^ = 0.144	F = 0.569, *p* = 0.452, partial η^2^ = 0.005
STAI-T	F = 0.019, *p* = 0.892, partial η^2^ < 0.001	F = 9.965, *p* = 0.002, partial η^2^ = 0.085	F = 0.636, *p* = 0.427, partial η^2^ = 0.006
AQ	F = 2.644, *p* = 0.107, partial η^2^ = 0.024	F = 8.105, *p* = 0.005, partial η^2^ = 0.070	F = 0.792, *p* = 0.376, partial η^2^ = 0.007
BIS	F = 1.955, *p* = 0.165, partial η^2^ = 0.018	F = 0.096, *p* = 0.757, partial η^2^ = 0.001	F = 0.653, *p* = 0.421, partial η^2^ = 0.006
PHCSCS	F = 0.420, *p* = 0.518, partial η^2^ = 0.004	F = 1.537, *p* = 0.218, partial η^2^ = 0.014	F = 4.143, *p* = 0.044, partial η^2^ = 0.037

Note. Significant Time effects were observed for SIQ-JR, FASM, CES-DC, STAI-T, and AQ. A significant Group × Time interaction was observed for PHCSCS only. *p* < 0.05 indicates statistical significance.

**Table 5:** ANCOVA Results controlling for baseline scores.

Variable	Group F	*p*	Partial η^2^	Adjusted Mean SPT	Adjusted Mean DBT	Significant
SIQ-JR	0.993	0.321	0.009	18.81	21.99	No
FASM	3.474	0.065	0.032	5.48	8.76	No
CES-DC	0.117	0.733	0.001	21.51	20.9	No
STAI-T	0.433	0.512	0.004	47.43	46.46	No
AQ	1.735	0.191	0.016	62.22	65.27	No
BIS	1.488	0.225	0.014	70.73	68.76	No
PHCSCS	4.327	0.040	0.039	108.97	115.42	Yes

Note. Only PHCSCS showed a significant group difference after controlling for baseline (*p* = 0.040). Adjusted means are estimated marginal means from ANCOVA models controlling for baseline scores.

**Table 6:** Within-group and between-group effect sizes.

Variable	SPT-SAFE Change	SPT d	SPT *p*	DBT-BI Change	DBT d	DBT *p*
SIQ-JR	−11.47 (19.81)	−0.518	<0.001	−9.36 (20.28)	−0.363	0.002
FASM	−5.36 (9.55)	−0.447	<0.001	−2.92 (12.65)	−0.225	0.109
CES-DC	−3.49 (9.38)	−0.306	0.006	−4.98 (11.23)	−0.344	0.003
STAI-T	−1.92 (7.93)	−0.240	0.069	−3.18 (8.62)	−0.305	0.012
AQ	−4.41 (13.29)	−0.313	0.014	−2.26 (11.61)	−0.132	0.175
BIS	0.37 (8.18)	0.046	0.728	−1.02 (9.81)	−0.097	0.466
PHCSCS	−1.05 (17.09)	−0.055	0.638	5.72 (17.56)	0.258	0.026

Note. *p* < 0.05 indicates statistical significance. Negative values indicate improvement for SIQ-JR, FASM, CES-DC, STAI-T, AQ, and BIS, whereas positive values indicate improvement for PHCSCS. Cohen’s d: small = 0.20, medium = 0.50, and large = 0.80.

## Data Availability

Due to the sensitive nature of the data involving children and suicide-related clinical information, access to the dataset is strictly controlled. De-identified data may be made available to qualified researchers upon reasonable request, following approval by the Institutional Review Board. Data will be provided solely for academic research purposes, and any secondary use requires explicit ethical approval. No identifiable information is included, and data sharing complies with all applicable privacy and data protection regulations.

## Abbreviations

The following abbreviations are used in this manuscript:

SPT-SAFE	Sandplay Therapy with Suicide- and Self-injury Focused Engagement
DBT-BI	Dialectical Behavior Therapy–informed Brief Intervention
NSSI	Non-Suicidal Self-Injury
C-SSRS	Columbia Suicide Severity Rating Scale
SIQ-JR	Suicidal Ideation Questionnaire–Junior
CES-DC	Center for Epidemiologic Studies Depression Scale for Children
STAI-T	State–Trait Anxiety Inventory for Children—Trait
PHCSCS	Piers–Harris Children’s Self-Concept Scale
FASM	Functional Assessment of Self-Mutilation
